# MMP-3 in the peripheral serum as a biomarker of knee osteoarthritis, 40 years after open total knee meniscectomy

**DOI:** 10.1186/s40634-018-0132-x

**Published:** 2018-06-15

**Authors:** Ioannis Pengas, Suzanne Eldridge, Aggelos Assiotis, Michael McNicholas, Joao Espregueira Mendes, Lior Laver

**Affiliations:** 1 Consultant Trauma & Orthopaedic Knee Surgeon, Joint Preservation & Soft Tissue Knee Specialist, Royal Cornwall Teaching Hospitals NHS Trust Treliske, Truro, TR1 3LQ UK; 2 Department of Experimental Medicine and Rheumatology, William Harvey Research Institute. Barts and The London, Queen Mary’s School of Medicine and Dentistry, London, EC1M 6BQ UK; 3 Specialist registrar in Trauma & Orthopaedics, Chelsea & Westminster Hospital NHS Foundation Trust, 369 Fulham Rd, Chelsea, London, SW10 9NH UK; 4 Consultant Trauma & Orthopaedic Knee Surgeon, Aintree University Hospital NHS Foundation Trust Longmoor Ln, Liverpool, L9 7AL UK; 5 Orthopaedics Department of Minho University, R. da Universidade, Minho University, 4710-057 Braga, Portugal; 6 Department of Arthroscopy, The Royal Orthopaedic Hospital, The Woodlands, Bristol Rd S, Birmingham, B31 2AP UK

## Abstract

**Background:**

To explore potential biomarkers in a meniscectomy-induced knee osteoarthritis model, at forty years after meniscectomy.

**Methods:**

We carried out a forty-year study of 53 patients who, as adolescents, underwent open total meniscectomy and assessed two potential synovial and serum biomarkers, namely glycosaminoglycan (GAG) and matrix metalloproteinase-3 (MMP-3). Of the 30 patients available for review, 8 had contralateral knee operations and were excluded.

Of the remaining 22 patients, 17 had successful operated knee synovial fluid aspirations and 8 also had successful contralateral control knee aspirations. GAG and MMP3 levels in the synovial fluid and peripheral serum was measured using Alcian blue precipitation and ELISA quantification, respectively. Patients also had their knee radiographs assessed and their radiographic osteoarthritis classified as per the Kellgren-Lawrence and Ahlbӓck systems.

**Results:**

At forty years after meniscectomy, synovial MMP-3 levels remain increased (*p* = 0.0132) while GAG levels were reduced (*p* = 0.0487) when compared to controls and these two levels correlate inversely. Furthermore, levels of synovial MMP-3 significantly correlated (*p* = 0.0032, *r* = 0.7734; *p* = 0.0256, *r* = 0.5552) and GAG levels significantly inversely correlated (*p* = 0.0308, *r* = − 0.6220; *p* = 0.0135, *r* = − 0.6024), respectively, with both radiological scoring systems. Interestingly, we found that the levels of serum MMP-3 correlated only with the synovial fluid levels of MMP-3 in the operated knee and not with the non-operated joint (*p* = 0.0252, *r* = 0.7706 vs. *p* = 0.0764, *r* = 0.6576). Multiple regression analysis for patient’s quality of life based on these biomarkers revealed an almost perfect result with an R2 of 0.9998 and a *p* value = 0.0087.

**Conclusion:**

Our results suggest that serum levels of MMP3 could be used as a potential biomarker for knee osteoarthritis, using a simple blood test. Larger cohorts are desirable in order to prove or disprove this finding.

**Electronic supplementary material:**

The online version of this article (10.1186/s40634-018-0132-x) contains supplementary material, which is available to authorized users.

## Background

Osteoarthritis (OA), along with Alzheimer’s disease, have been characterised as “high burden diseases with no curative treatments” by the World Health Organization (WHO) as they both lack an identifiable biomarker (Kaplan, 2004).

OA is a severely disabling disease which affects a third of the population over 50 years worldwide, resulting in major socio-economic burdens (Goldring et al., 2006; Kinds et al., 2011). Aging and mechanical injury to the joint are the primary causes of OA; however the symptoms associated with OA are often not displayed until months or years following a physical insult (Swiontkowski et al., 2000; Vincent & Saklatvala, 2008). Common knee injuries include direct post-traumatic injury to the articular cartilage, altered knee biomechanics from cruciate ligament rupture or meniscal loss or limb malalignment; resulting in disruption of joint biomechanics and the joints homeostatic pathways within the articular cartilage extracellular matrix (ECM), which in turn leads to irreversible cartilage destruction (Sherwood et al., 2014).

Despite the short-medium term relief from joint instability and discomfort, it has been suggested that partial or total meniscectomy (affecting 61/100,000 of the population per year) (Baker et al., 1985) has detrimental effects that eventually lead to symptomatic and radiological knee OA in up to 50% of patients within 5–15 years and increased risk of requiring knee arthroplasty in the future (Pengas et al., 2012a; Appel, 1970; Jorgensen et al., 1987; Hede et al., 1992).

Radiological assessment of joint osteoarthritis relies upon plain films as the gold standard investigation with high resolution Magnetic Resonance Imaging (MRI) assessment as a potential alternative (Eckstein et al., 2006). It is evident that these investigations demonstrate established disease, inadequate for early identification of the condition, which does not always correlate with patients’ perception of symptoms or progression of the condition (Lohmander, 2004; Lawrence et al., 1966; Hannan et al., 2000). This may be attributed to the avascular and aneural nature of articular cartilage, suggesting that patients presenting with symptomatic osteoarthritis often have significant and irreparable cartilage damage, ultimately resulting in knee arthroplasty as a definitive solution.

In order to achieve an early and timely identification of the disease, patient reported outcome measures (PROMs) and biomarkers in isolation or in combination with radiographs have been suggested as possible diagnostic adjuncts. It would be invaluable to patients and healthcare systems if it were possible to screen high risk patients with a biomarker investigation. The success of biomarkers used in this context would be dependent on their ability to indicate the onset of this biological process and in theory respond to interventions in a timely manner (De Gruttola et al., 2001). Several molecules have been proposed as possible biomarkers of OA two of these being glycosaminoglycans (GAGs) and matrix metalloproteinases (MMPs).

Proteoglycans (Nagase & Matrix Metalloproteinases, 1999) are produced by chondrocytes as large hydrophilic negatively charged polysaccharides responsible for the compressive strength of articular cartilage. Increased levels of MMPs have been detected following knee injury and osteoarthritis, as these degradative enzymes play a key role in the proteolysis of cartilage matrix molecules, including proteoglycans (Nagase & Matrix Metalloproteinases, 1999; Lohmander et al., 1994; Lohmander et al., 1999). Proteolysis of proteoglycans, releasing glycosaminoglycan-containing fragments (GAGs), is an early and critical feature of cartilage breakdown seen following injury such as one involving the meniscus (Liu et al., 2017) or primary OA (Lohmander et al., 1989) and it has been demonstrated that both GAGs and MMPs are elevated in the synovial fluid of osteoarthritic knees (Lohmander et al., 1999; Lohmander et al., 1989; Chu et al., 2015).

In this study, the serum and synovial fluid levels of two biomarkers are reported and compared to the non-operated knee at a mean 40 year follow-up of a cohort of patients that underwent open total meniscectomies as adolescents with otherwise pristine knees under the care of a single surgeon. With abundant evidence in the literature indicating that total meniscectomy leads to joint degeneration, increased incidence of knee OA and need for arthroplasty procedures (Pengas et al., 2012b; Hoser et al., 2001; Englund & Lohmander, 2004; Rockborn & Gillquist, 1996; Lee et al., 2006). Previous analysis of this cohort revealed a > 4 fold increase in knee OA and 132 fold increase in knee arthroplasty. An association has also been established between the cohort’s OA radiographic findings and recorded range of motion and PROMS (Pengas et al., 2012b), it follows that an attempt to evaluate biomarkers for OA in this cohort is logical.

Our hypothesis is that there is a significant difference in MMP-3 and GAG levels between the operated and non-operated knees which correlates with their serum levels, potentially allowing their use as biomarkers in tracking disease progression in this model of knee osteoarthritis.

## Methods

### Patient selection criteria

Under the care of the late Professor Ian Smillie, 313 adolescent patients underwent open total meniscectomy. One hundred of these patients that were identified as not having any other intra-articular knee pathology at the time of the operation were reviewed at 17 and 30 years post-operatively (Abdon et al., 1990; McNicholas et al., 2000). At the 30-year follow-up, both knees were evaluated radiographically in 53 patients for whom an ethical approval to be assessed at a mean of 40 years post operatively was obtained.

At the time of review, 7 patients had undergone a total knee replacement, 5 had passed away, 6 were lost to follow-up and a further 5 were unable to attend for clinical evaluation. Of the 30 patients available for clinical review, 8 had contralateral knee operations and were excluded as that knee would not be able to be used as a control. This resulted in 22 suitable patients for our study with no other knee intervention other than the removed meniscus.

All of the suitable patients (*n* = 22) had blood collected into 3 plain 6 mL EDTA vacutainer tubes for serum MMP-3 level quantification. This was inverted 8 times and allowed to clot at room temperature.

Patients also underwent operated and non-operated knee aspirations. Seventeen (*n* = 17) yielded a successful operated knee synovial fluid aspirate and out of those, eight (*n* = 8) yielded a successful contralateral control knee aspirate (please see addendum tables depicting the cohorts demographics, radiological, clinical, PROMs and biomarkers raw values). We were not able to increase our non-operated knee aspirate samples as knee washout-aspiration was not granted ethical approval and therefore was not performed. Aspirate samples were collected undiluted and transferred in plain tubes.

Samples were transferred to the on-site biochemistry laboratory and were centrifuged. They were labelled and stored at − 70 °C and safeguarded against repetitive freeze-thawing cycles whilst awaiting transfer for analysis to the biochemical laboratory at Lund University, Sweden.

### MMP-3 quantification

MMP-3 values were determined by a stromelysin-trapping, enzyme-lined immunosorbent assay (Walakovits et al., 1992). MaxiSorp surface (96-well) plates (Nunc, Roskilde, Denmark) were coated overnight at 4 °C with 100 l/well of a 1.0 g/ml solution of the murine stromelysin anti-dog monoclonal antibody MAC085 (Celltech, Slough, UK) in phosphate buffered saline (PBS) coating solution (KPL, Gaithersburg, MD). The plates were then washed 4 times in a solution of 2 m*M* of imidazole-buffered saline and 0.02% Tween 20 (KPL, Gaithersburg, MD) and incubated for 20 min at room temperature with 1% bovine serum albumin (BSA) in PBS (BSA blocking solution; KPL) to block nonspecific protein binding to the wells.

Subsequently the BSA was washed from the plates and standard recombinant human prostromelysin (Merck, Rahway, NJ) or samples of plasma were added (100 l/well) for 1 h at room temperature. The samples were then diluted with 0.67% BSA in PBS (BSA diluent solution; KPL). The plates were washed and then incubated with a 10 g/ml solution of rabbit polyclonal anti-human stromelysin IgG (Merck) in BSA diluent solution. The plates were washed again, after which they were incubated for 1 h at room temperature with peroxidase-labelled goat anti-rabbit IgG (KPL) diluted to 125 ng/ml in BSA solution (100 l/well). The plates were washed again and were incubated for 5 min with a etramethylbenzidine (TMB)– hydrogen peroxide (H_2_O_2_) solution (0.2 g/litre TMB, 0.01% H_2_O_2_), after which the reaction was stopped by the addition of 1 *M* phosphoric acid. Absorbance at 450 nm was measured spectrophotometrically using a Multiscan Multisoft plate reader (Labsystems, Helsinki, Finland) and the software Ascent 2.4.2 (Thermo Electron, Waltham, WA).

### GAG quantification

Concentration of GAG was measured by a modified Björnsson Alcian blue precipitation (Bjornsson, 1993). This measures Alcian blue dye binding in proportion to the number of negative charges on GAG chains. Samples and chondroitin sulfate standards (25 μl) were precipitated for two hours at 4 °C with 0.04% *w*/*v* Alcian blue, 0.72 M guanidinium hydrochloride, 0.25% w/v Triton X − 100, and 0.1% *v*/v H_2_SO_4_ (0.45 ml). The precipitates were collected after centrifugation (16,000 g, 15 min, 4 °C), then dissolved in 4 M guanidinium hydrochloride, 33% v/v 1-propanol (0.25 ml), and transferred to 96-well micro-titer plates prior to absorbance measurement at 600 nm (Larsson et al., 2009).

### Radiographic analysis

Radiographic analysis included bilateral knee antero-posterior weight-bearing radiographs, with the knee flexed at 15°. These radiographs were blindly and independently scored by two investigators (IP, AA) from a distance of 60 cm without magnifying equipment, using the Kellgren-Lawrence (Abdon et al., 1990) and the Ahlbäck (McNicholas et al., 2000) scoring systems for knee tibiofemoral joint (TFJ) OA. These are two of the most widely accepted radiographic scoring systems for knee OA.

### Statistical analysis

Data were analysed using GraphPad Prism 6 (GraphPad Software, Inc., California, USA). The Shapiro-Wilk test was used to test data for normality. Parametric data were compared using a paired t-test analysis. Correlations were analysed using the Pearson’s rank correlation coefficient. *P*-values < 0.05 were considered statistically significant. (* denoting *P* < 0.05, ** denoting *P* < 0.005, *** denoting *P* < 0.0005).

### Serum MMP-3 levels can predict MMP-3 levels in the synovial fluid of post-meniscectomy patients

**Fig. 1:**
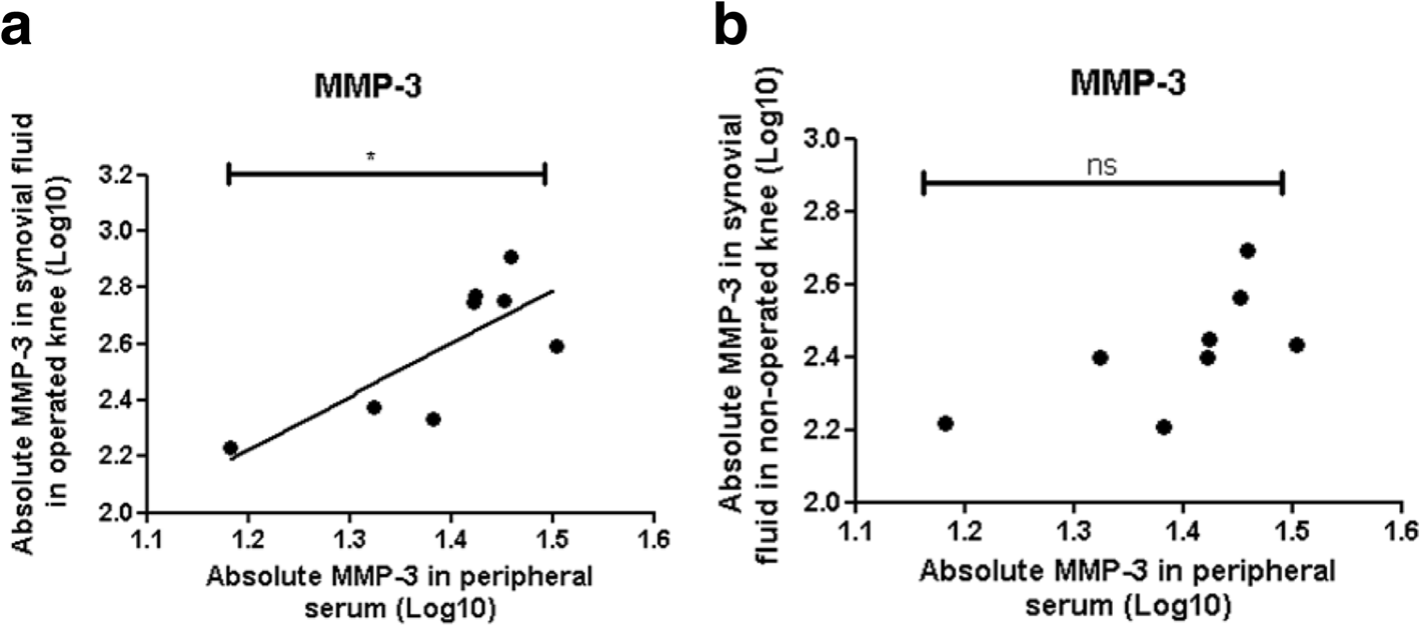
The levels of MMP-3 in the peripheral serum correlates directly with the levels in the synovial fluid of the operated but not non-operated knee joints. **a** MMP-3 protein levels in the peripheral serum plotted against the levels within the synovial fluid of the operated or **b** non-operated knee joints (*n* = 8)

Positive correlation was observed between the serum MMP-3 and synovial MMP-3 concentrations of the operated joints (*p* = 0.0252, *r* = 0.7706) (Fig. 1a) but not with the MMP-3 concentrations of the non-operated joints (*p* = 0.0764, *r* = 0.6576) (Fig. 1b). Serum MMP-3 levels could therefore potentially be utilised as a surrogate marker of synovial MMP-3 levels in these operated knees.

## Results

### *MMP-3 is chronically upregulated and correlates with decreased GAG at* 40 years post-meniscectomy

**Fig. 2:**
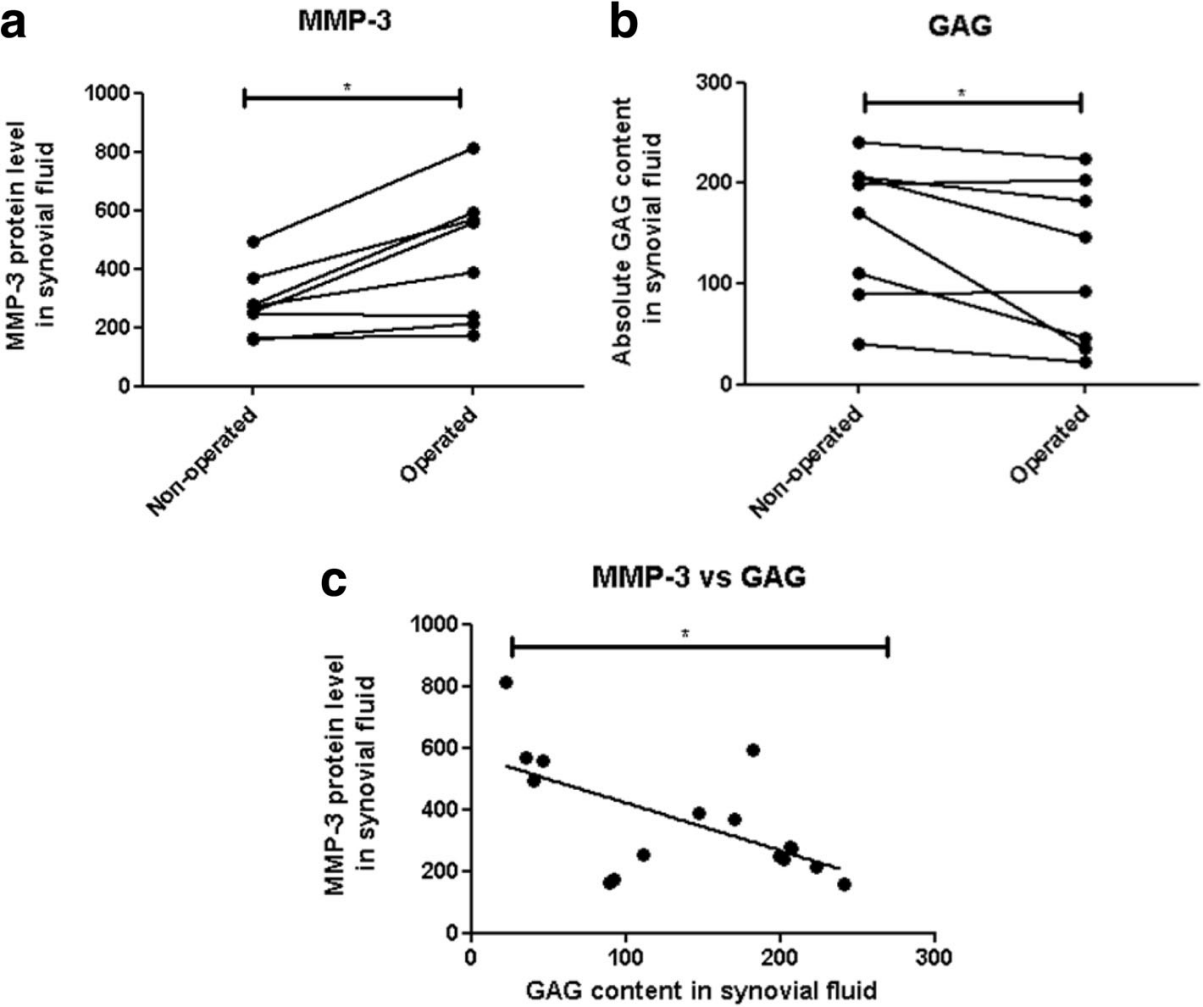
MMP-3 is chronically upregulated and correlates with decreased GAG content in the synovial fluid of the operated knee in patients 40 years after meniscectomy. **a** MMP-3 and **b** GAG protein levels within the synovial fluid of patients 40 years post total meniscectomy in operated or non-operated knees (*n* = 8). **c** Correlation of absolute GAG levels plotted against absolute MMP-3 levels in the synovial fluid all knees (*n* = 16)

We compared levels of MMP-3 and GAGs in the operated and non-operated knees of the 8 patients that had successful bilateral knee synovial fluid aspirations. Paired analysis of the levels of MMP-3 in the synovial fluid revealed that even at this late time point after surgery, the levels of MMP3 are significantly higher in the operated knee (*p* = 0.0132) (Fig. 2a), with synovial GAGs levels in the operated joint significantly reduced (*p* = 0.0487) (Fig. 2b). There was an inverse correlation between the levels MMP-3 and GAGs levels within the synovial fluid when comparing all 16 samples from both operated and non-operated knees (*p* = 0.0458, *r* = − 0.73810) (Fig. 2c). The mean values and standard deviations of GAG and MMP-3 concentrations are presented in Table 1.

**Table 1:** Unoperated and operated mean values and standard deviations of MMP-3 protein and GAG content in the synovial fluid

Measured outcome	Unoperated Mean ± SD (Range)	Operated Mean ± SD (Range)	*p* Value
MMP3 content	279.91 ± 108.57	443.08 ± 226.11	0.0132
	(160.7–493.49)	(171.28–812.3)	
GAG content	158.18 ± 70.01	119.21 ± 80.31	0.0487
	(401.3–241.1)	(22.28–223.87)	

### Increased synovial MMP-3 and reduced GAG levels correlate with radiographic scores

**Fig. 3:**
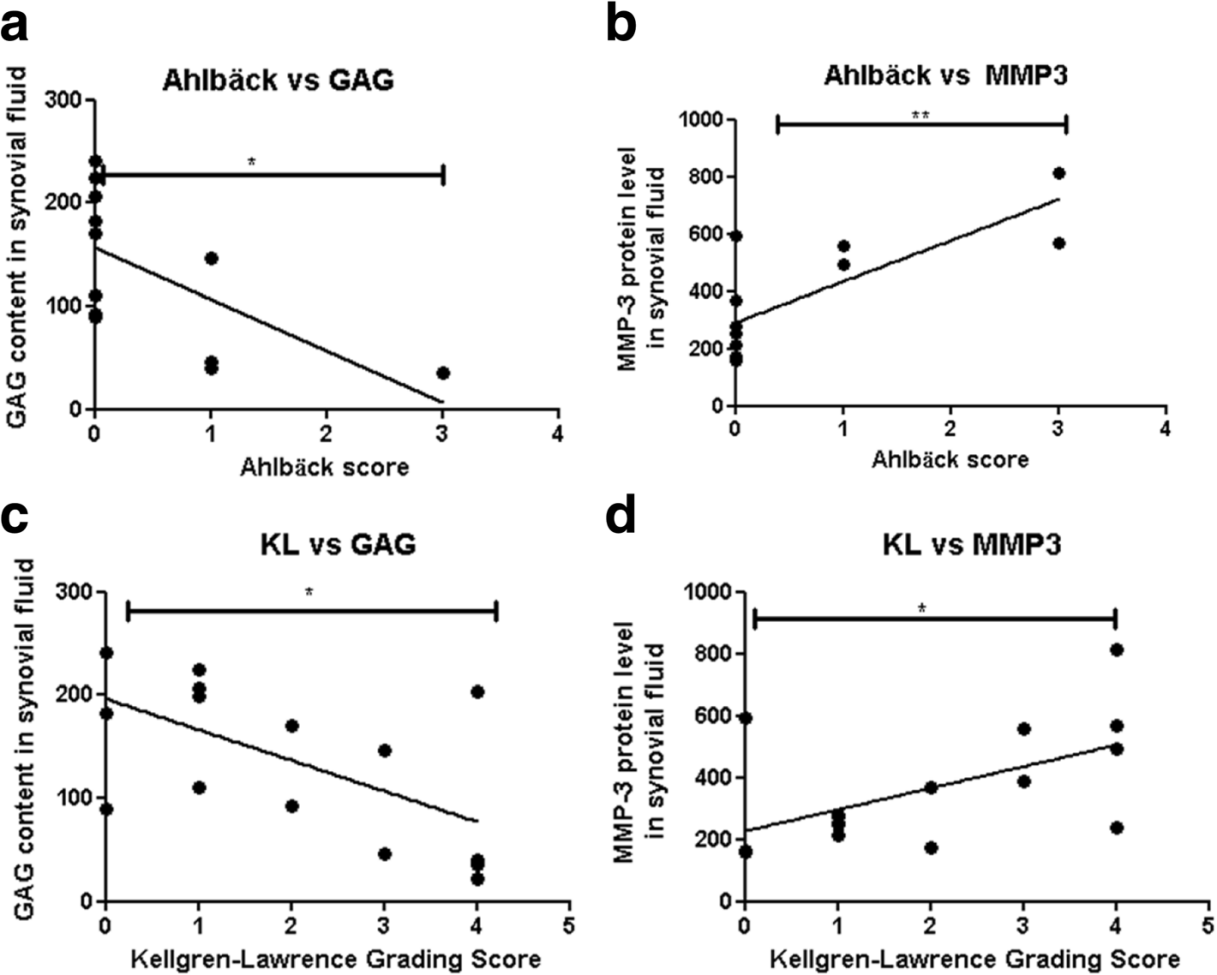
MMP-3 and GAG levels correlate with Ahlbӓck and Kellgren-Laurence (KL) radiographic scores. **a** Ahlbӓck vs GAG; **b** Ahlbӓck vs MMP-3; **c** Kellgren-Laurence vs GAG; and **d** Kellgren-Laurence vs MMP-3 levels in the synovial fluid of all knees (*n* = 16)

Operated knee synovial levels of MMP-3 correlated and GAG inversely correlated significantly with both radiographic evaluation systems of Ahlbӓck (*p* = 0.0032, *r* = 0.7734; *p* = 0.0308, *r* = − 0.6220) (Fig. 3a and b respectively) and Kellgren-Laurence (KL) (*p* = 0.0256, *r* = 0.5552; *p* = 0.0135, *r* = − 0.6024) (Fig. 3c and d respectively).

### GAG levels, patient age and MMP-3 levels predict quality of life in a multiple regression model

**Fig. 4:**
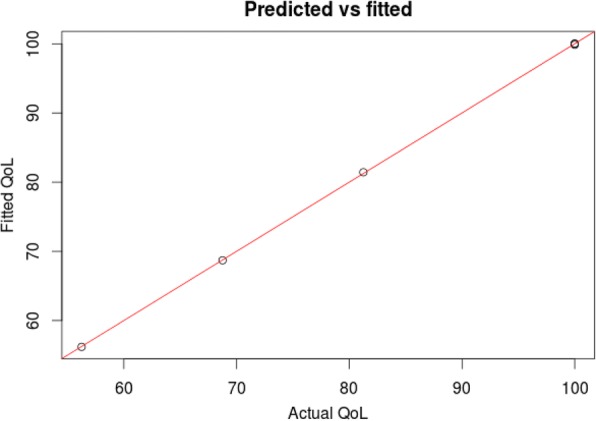
Multiple regression model incorporating patient GAG levels pre- and post-surgery and MMP3 levels post-surgery to predict patient quality of life. The model shows quality of life (QoL) fits with an R^2^ of 0.9998 and a *p* value = 0.0087

To understand how these parameters affect quality of life 40 years after meniscectomy, we built a mathematical model in which, using clinical and biochemical parameters we could predict the quality of life using a stepwise regression (AIC function in R) (Sakamoto & information, 1986). The final model included age at surgery, GAG 40 years after surgery, the change in GAGs from baseline and the amount of the MMP-3 levels 40 years after surgery. The coefficients are displayed in (Fig. 4) and (Table 2) and the R script and diagnostic plots are shown in Additional file 1. Remarkably this model predicted the quality of life (QOL) almost perfectly, with an R^2^ of 0.9998 and a *p* value = 0.0087. Taken together, these data suggest that these factors all contribute to quality of life.

**Table 2:** Coefficients of multiple regression analysis for patient quality of life

	Estimate	Std. Error	t value	Pr(>|t|)
(Intercept)	-1.41E + 02	4.85E + 00	−29.16	0.02182*
AgeAtSur	1.15E + 01	2.97E-01	38.61	0.01648*
Gagind40	3.92E-01	3.67E-03	106.92	0.00595**
dGAG40	−2.10E + 00	2.75E-02	−76.48	0.00832**
MMP3ind40	−1.55E-01	2.48E-03	−62.63	0.01016*

Signif. codes: 0.001 ‘**’ 0.01 ‘*’ 0.05 ‘.’ 0.1 ‘’ 1

Residual standard error: 0.2449 on 1 degrees of freedom

Multiple R-squared: 1, Adjusted R-squared: 0.9998

F-statistic: 7406 on 4 and 1 DF, *p*-value: 0.008715

## Discussion

The most interesting finding of this study is that synovial MMP-3 levels are upregulated in operated knees, correlating with radiographic scores of OA and inversely correlating to the drop in synovial GAG concentration. The fact that MMP-3 levels were raised so long after the cartilage insult indicates the presence of a chronic response and not a short lived inflammatory one as previously suggested. Interestingly, operated knee synovial MMP-3 levels correlated with serum MMP-3 levels, whereas non-operated knee synovial MMP-3 levels did not. This is an important finding, as it presents serum MMP-3 levels as a potential and easily measurable biomarker for knee OA.

In addition, multiple regression analysis demonstrated that we can measure the patient’s quality of life based on these biomarkers, which unlike PROMs cannot be “manipulated or exaggerated” by the patients, providing a much more robust measurement which can also allow us to estimate the QoL a patient would likely have after surgery.

This study also demonstrated that synovial fluid GAG levels were reduced in operated knees compared to non-operated knees and were inversely correlated to both Kellgren-Laurence and Ahlbӓck radiographic scores of OA. This study confirms that the greater the interval between meniscectomy, and synovial fluid sampling, the lower the concentration of GAG observed.

In OA, a number of proteases generated by the synovial membrane and chondrocytes damage the molecular and architectural structure of cartilage. They cause degradation of cartilage matrix and result in the generation of molecular fragments which are subsequently released from cartilage into the synovial fluid and into the systemic circulation. Two of these hydrolysed molecules are type-II collagen and aggrecan (Jones & Riley, 2005). Early matrix damage and acute injury have been associated with increased aggrecan proteolysis and increased levels of proteoglycan fragments in the synovial fluid which however were seen to decline with time (Lohmander et al., 1999; Lohmander et al., 1989; Pratta et al., 2003; Dahlberg et al., 1992; Lohmander et al., 1992; Lohmander et al., 1993; O’Driscoll, 1998; Rothwell & Bentley, 1973). A finding supported by Larsson et al. in a mean 18 years follow-up post meniscectomy, measuring aggrecanase-generated ARGS neopeptite study (Larsson et al., 2010; Larsson et al., 2012).

When examining proteases, it seems that stromelysin (MMP-3) and collagenase (MMP-1) are two of the most extensively studied biomarkers (Poole, 2003). MMP-3 is produced by synovial membrane cells and by chondrocytes in response to mechanical stimulation and exposure to inflammatory cytokines (Fitzgerald et al., 2004). Although not all investigators agree that there is significant increase in the levels of MMP-3 or MMP-1 in patients with OA (Garnero et al., 2005), increased levels of synovial fluid MMP-3 have been detected in patients suffering with hip and knee OA (Chen et al., 2014; Ulrich-Vinther et al., 2003; Georgiev et al., 2018) and in smaller joint degeneration (Jiang et al., 2013), exhibiting greater increases as compared with MMP-1 levels (Lohmander et al., 1993; Ishiguro et al., 1999). Recently, elevated MMP-3 levels were highlighted in post-menisectomy subjects (Liu et al., 2017), suggesting that it would be reasonable to consider synovial fluid MMP-3 as a potential biomarker for OA and perhaps a valid biomarker for follow up in post-menisectomy patients.

We selected a model of knee OA on the background of total meniscectomy as there is strong evidence in the literature that total meniscectomy leads to joint degeneration resulting in increased incidence of knee osteoarthritis (Pengas et al., 2012b; Hoser et al., 2001; Englund & Lohmander, 2004; Rockborn & Gillquist, 1996; Lee et al., 2006). Hence, one of the strong suits of this study lies in the very well defined and studied cohort of patients. All patients in this cohort underwent open meniscectomy with a uniform operative technique and post-operative rehabilitation protocol and their follow-up period of 40 years is unique in the published literature.

The use of biomarkers as surrogate endpoints in the diagnosis of OA is understandably desirable to both scientists and surgeons alike, however it is acknowledged that these molecules are metabolised by other tissues and serum levels may be affected by this; oversimplification of their metabolic pathways should be avoided and interpretation should be made with caution (Myers, 1999). Arguably the small number of subjects in this truly long term follow-up study limits our ability to reach concrete conclusions that can be generalised; however we have indicated that MMP-3 has the potential of being utilised as a marker although further research is indicated.

An improved approach for assessment of disease progression in OA could be achieved via a ‘multisource feedback’, combining radiographic evaluation with PROMs and biomarkers such as MMP-3, as the quest for a simple and easily measurable OA biomarker continues. The fact that this has not yet fully materialised serves to underline the complexity of this ‘simple’ monocellular tissue.

## Conclusions

Our results suggest that serum levels of MMP3 could be used as a potential biomarker for knee osteoarthritis and possible disease predictor, using a simple blood test. Larger cohorts are desirable in order to prove or disprove this finding.

## Additional file

Additional file 1: (R plots) & Addendum. (DOC 164 kb)
