# Supplementary material for: MMP-3 in the peripheral serum as a biomarker of knee osteoarthritis, 40 years after open total knee meniscectomy
Source: J Exp Orthop. 2018 Jun 15;5:21. doi: 10.1186/s40634-018-0132-x (PMC6003895; doi:10.1186/s40634-018-0132-x)
Supplement: Supplementary file 1 — (R plots) & Addendum. (DOC 164 kb) [file 40634_2018_132_MOESM1_ESM.doc]

**Supplementary figure 1. Multiple regression analysis residual plots and R script.**

Load the data

>attach(crude.data1)

#Fit the model with all the variables

>fit<-lm(QOL40 ~ AgeAtSur + age + Gagind40 + dGAG40 + MMP3ind40)

#reduce the variables

>library(MASS)

>step <- stepAIC(fit, direction="both")

Start: AIC=-17.63
QOL40 ~ AgeAtSur + age + Gagind40 + dGAG40 + MMP3ind40


Step: AIC=-17.63
QOL40 ~ AgeAtSur + Gagind40 + dGAG40 + MMP3ind40 #this is the new model that's just as good.

Df Sum of Sq RSS AIC
<none> 0.06 -17.631
- AgeAtSur 1 89.45 89.51 24.215
- MMP3ind40 1 235.34 235.40 30.017
- dGAG40 1 350.90 350.96 32.413
- Gagind40 1 685.84 685.90 36.434

#repeat the fit with the second model

> fit <- lm(QOL40~AgeAtSur+Gagind40+dGAG40+MMP3ind40,data=crude.data1)
> summary(fit)

Call:
QOL.fit<-lm(formula = QOL40 ~ AgeAtSur + Gagind40 + dGAG40 + MMP3ind40,
 data = crude.data1)

Residuals:
 1 2 3 4 5 6
-0.183764 0.142616 0.048506 0.040430 -0.043372 -0.004416

Coefficients:
 Estimate Std. Error t value Pr(>|t|)
(Intercept) -1.413e+02 4.847e+00 -29.16 0.02182 *
AgeAtSur 1.146e+01 2.969e-01 38.61 0.01648 *
Gagind40 3.919e-01 3.665e-03 106.92 0.00595 **
dGAG40 -2.103e+00 2.750e-02 -76.48 0.00832 **
MMP3ind40 -1.553e-01 2.479e-03 -62.63 0.01016 *
---
Signif. codes: 0 ‘***’ 0.001 ‘**’ 0.01 ‘*’ 0.05 ‘.’ 0.1 ‘ ’ 1

Residual standard error: 0.2449 on 1 degrees of freedom
Multiple R-squared: 1, Adjusted R-squared: 0.9998
F-statistic: 7406 on 4 and 1 DF, p-value: 0.008715

Plot the fitted vs experimental data

> fitted.qol<-QOL.fit$fitted.values
#Plot predicted vs fitted with regression line

> abline(lm(QOL40~fitted.qol), col="red")

> plot(fit)


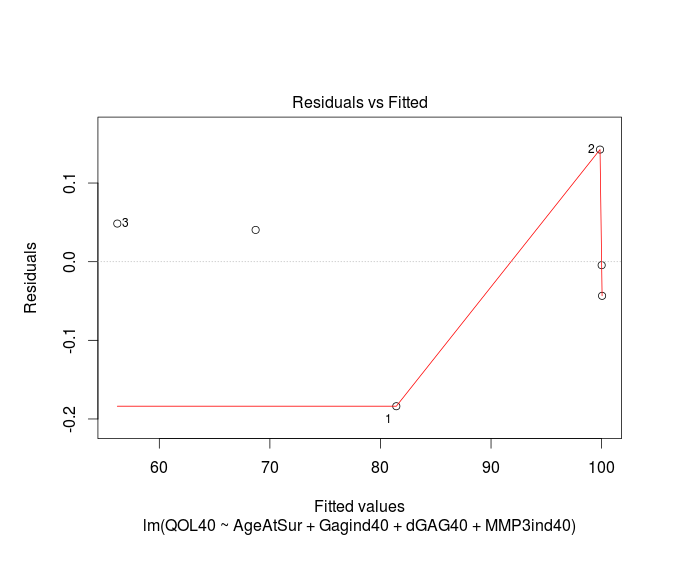


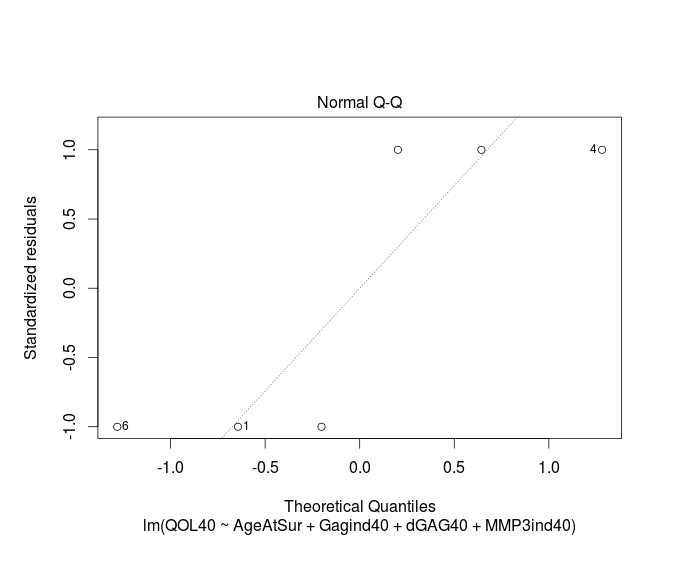


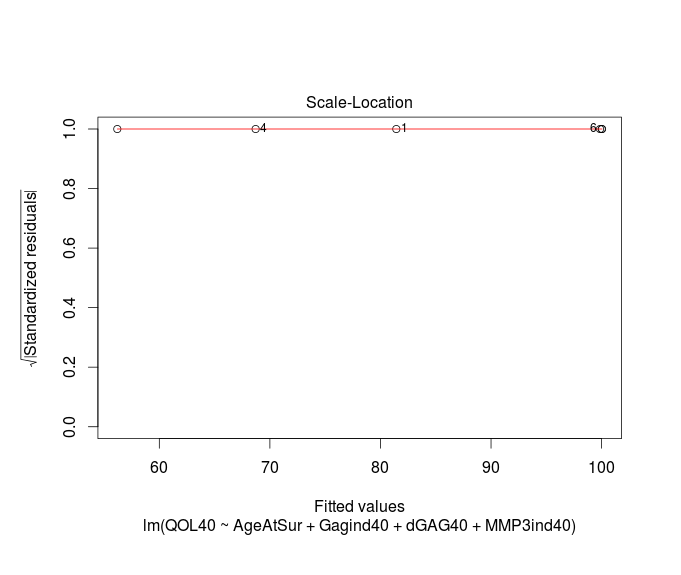


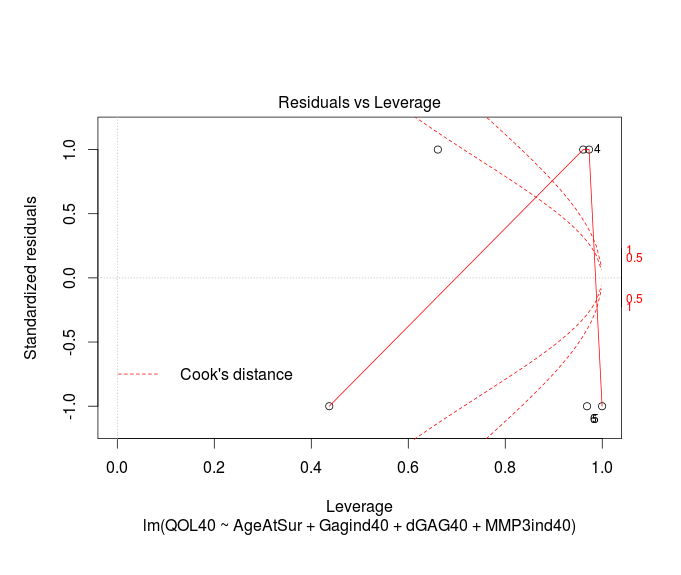


Next, to understand how these parameters affect quality of life 40 years after meniscectomy, we built a mathematical model in which, using clinical and biochemical parameters we could predict the quality of life using a stepwise regression (AIC function in R{ Sakamoto, Y., Ishiguro, M., and Kitagawa G. (1986). *Akaike Information Criterion Statistics*. D. Reidel Publishing Company.}). The final model included age at surgery, GAG 40 years after surgery, the change in GAGs from baseline and the amount of the MMP-3 levels 40 years after surgery. The coefficients are displayed in figure 4. Remarkably this model predicted the QOL almost perfectly, with a R^2 of 0.9998 and a p value = 0.0087. Taken together, these data suggest that these factors all contribute to quality of life.

The R script and diagnostic plots are shown in supplementary materials.

| Age at Meniscectomy | BMI  At review | Occupation  At review | Total Meniscectomy Site | Side | GAG-Index knee | GAG-Non-Index | MMP-3 Index | MMP-3 Non-Index | MMP-3 SERUM (Elisa) | KS-OA1 | ROM  Index knee  Hyper- Extension | ROM  Index Knee  Max flexion | ROM Non-Index Hyper extension | ROM  Index Knee  Max flexion | Ahlbäck  INDEX KNEE | KL  INDEX KNEE | Ahlbäck Non-INDEX KNEE | KL  NON-INDEX KNEE |
| --- | --- | --- | --- | --- | --- | --- | --- | --- | --- | --- | --- | --- | --- | --- | --- | --- | --- | --- |
| **18** | 35.9 | Care Taker | **MM** | Right | 22.28 | 40.1 | 493.49 | 812.3 | 28.8 | < min | -15.0 | 90.0 | -10.0 | 100.0 | 3 | 4 | 1 | 4 |
| 17 | 28.3 | Print Broker | **MM** | Right | 46.6 | 111.2 | 559.02 | 251.5 | 26.5 | < min | -5.0 | 130.0 | 0.0 | 140.0 | 1 | 3 | 0 | 1 |
| **16** | 31 | Electrician | **MM** | Left | 202.85 | 199.7 | 237.1 | 249.3 | 21.1 | < min | -5.0 | 125.0 | 0.0 | 130.0 | 3 | 4 | 1 | 1 |
| **16** | 32.7 | PE Teacher | **MM** | Right | 147.07 | 207.1 | 390.56 | 272.2 | 31.9 | < min | -10.0 | 125.0 | 10.0 | 125.0 | 1 | 3 | 0 | 1 |
| **18** | 26.2 | Sales Adviser | **LM** | Left | 182.86 | 206.3 | 591.77 | 280.7 | 26.6 | < min | 0.0 | 140.0 | 0.0 | 140.0 | 0 | 0 | 0 | 1 |
| **17** | 29.9 | Property Inspector | **LM** | Right | 92.6 | 89.6 | 171.28 | 164.5 | 15.2 | < min | 0.0 | 130.0 | 10.0 | 140.0 | 0 | 2 | 0 | 0 |
| **15** | 24.6 | Farmer | **LM** | Left | 223.87 | 241.1 | 215.82 | 160.7 | 24.1 | < min | -10.0 | 135.0 | 5.0 | 140.0 | 0 | 1 | 0 | 0 |
| **13** | 33.4 | Town Planner | **LM** | Left | 35.55 | 170.3 | 566.8 | 366.9 | 28.3 | < min | -5.0 | 125.0 | 0.0 | 130.0 | 3 | 4 | 0 | 2 |

Addendum:

| Age at Meniscectomy | BMI  At review | Occupation  At review | Total Meniscectomy Site | Side | KOOS | | | | | | | | IKDC  RAW | IKDC  SCORE |
| --- | --- | --- | --- | --- | --- | --- | --- | --- | --- | --- | --- | --- | --- | --- |
|  |  |  |  |  | SYMPTOMS | PAIN | | ADL | | SPORT | | QOL |  |  |
| **18** | 35.9 | Care Taker | **MM** | Right | 14.29 | | 38.89 | | 69.12 | | 55.00 | 25.00 | 60 | 48.27586 |
| 17 | 28.3 | Print Broker | **MM** | Right | 75.00 | | 88.89 | | 85.29 | | 65.00 | 93.75 | 72 | 62.06897 |
| **16** | 31 | Electrician | **MM** | Left | 82.14 | | 75.00 | | 55.88 | | 60.00 | 81.25 | 72 | 62.06897 |
| **16** | 32.7 | PE Teacher | **MM** | Right | 92.86 | | 100.00 | | 100.00 | | 100.00 | 100.00 | 79 | 70.11494 |
| **18** | 26.2 | Sales Adviser | **LM** | Left | 89.29 | | 94.44 | | 100.00 | | 100.00 | 68.75 | 78 | 68.96552 |
| **17** | 29.9 | Property Inspector | **LM** | Right | 82.14 | | 91.67 | | 92.65 | | 50.00 | 56.25 | 70 | 59.77011 |
| **15** | 24.6 | Farmer | **LM** | Left | 96.43 | | 97.22 | | 100.00 | | 100.00 | 100.00 | 85 | 77.01149 |
| **13** | 33.4 | Town Planner | **LM** | Left | 100.00 | | 100.00 | | 100.00 | | 100.00 | 100.00 | 83 | 74.71264 |
